# Complementary and Alternative Medicine for Respiratory Tract Infectious Diseases: Prevention and Treatments

**DOI:** 10.1155/2014/913095

**Published:** 2014-06-12

**Authors:** Taixiang Wu, Zhao-Xiang Bian, Minawaer Abudu, Denise Adams, Seong-Gyu Ko

**Affiliations:** ^1^Chinese Clinical Trial Registry, West China Hospital, Sichuan University, China; ^2^School of Chinese Medicine, Hong Kong Baptist University, Hong Kong; ^3^The First Hospital, Xinjiang University, China; ^4^Department of Pediatrics, University of Alberta, Canada; ^5^Kyung Hee University, Seoul, Republic of Korea

Respiratory tract infectious diseases are a group of the most common, widespread diseases, from common cold and influenza to tuberculosis and pneumonia; some of them may cause serious and even life-threatening health problems. Most cases of upper respiratory tract infectious diseases include common cold and influenza, which are mainly caused by various virus infections, and tuberculosis, pneumonia, and other low respiratory tract infectious diseases caused by different bacterial infections.

Releasing the symptoms and preventing complicated bacterial infection are a rule of treating virus infected upper respiratory tract infectious diseases. Traditionally, herbal medicines are used to treat and prevent the respiratory tract infectious diseases such as common cold and influenza worldwide. For example, more than two hundred patent products of traditional Chinese medicines have been approved into market for treatment and prevention of the common cold as well as influenza. In the past decades, some herbal medicines are developed as injections for clinical use for treating respiratory tract infections, such as Caihu injection, Shuanghuanglian injection, Yuxincao injection, and Qinkailing injection. Some of these medicines are believed to effectively release the symptoms; some are believed to strengthen the immune function of patient and therefore result in the virus and bacteria being eliminated; some are believed to kill the microorganisms directly.

A fact is that although a lot of herbal medicines are widely used clinically, most evidence supporting their use came from poorly reported published clinical studies or studies with questionable methodology, as Drs. Q. Yang, H. Zhang, C. Zou, and L. Wu, concluded in their systematic reviews. Call for high quality evidence is a mission of this special issue.

Fortunately, Dr. J. Chang and her colleagues contributed high quality evidence about a patent Chinese medicine Shi-Cha capsule for common cold by a well-designed, well-conducted, and well-reported randomized controlled trial. In this multicenter study, they have done very well on the ethicas issues including protocol aproval by ethical committee, properly informed consent and registered their protocol in a public clinical trial registry—Chinese Clinical Trial Registry; and high quality methodological issues, including the randomization method and allocation procedure, the recruitment of patients, the data management, the analysis method of results, all of these were described in detail and very clearly according to CONSORT statement. This transparency makes readers confident to trust the result of the study and we believe such evidence should be graded as high quality. if the reporting referes to CONSORT for TCM to provide more information of the principle of using Shi-Cha capsule for with “wind-cold type common cold” will makes readers better understanding on the rational of this medicine. Another advantage of this trial is that placebo control was used for comparison with Shi-Cha capsule. Usually, use of a medicine in which “the effect is accepted by public recognition” as the control is one of rules of the new medicine development clinical trial in China. The shortcoming of this rule is that it caused difficulty to assess the effects for a lot of traditional Chinese medicines because of the lack of evidence of effect of the controls. For the self-limited disease, a placebo control should be encouraged to be used in the treatment study. We believe that this study and its report could be a good example of high quality study and good reporting.

Another valuable thing we have seen is that Dr. C. Zou and his colleagues, physicians of traditional Chinese medicine, are using evidence-based medicine (EBM) method to critically appraise the evidence and using the results to guide their clinical practice successfully. This absolutely is a revolutionary event in the field while most traditional medical clinicians may not know EBM exactly. We hope more and more clinicians in the field can use EBM method to guide their practices and make the health care service better and better.

Studies came from France, Japan, South Africa, and Taiwan provide from in vitro and animal study suggesting that some herbal medicines may have antimicrobial activity or can strengthen the immune function. It is expected that these lines of evidence will result in more confidence to support the clinical use of complementary and alternative medicines.

*Taixiang Wu* *Taixiang Wu* *Zhao-Xiang Bian* *Zhao-Xiang Bian* *Minawaer Abudu* *Minawaer Abudu* *Denise Adams* *Denise Adams* *Seong-Gyu Ko* *Seong-Gyu Ko*

